# Non-celiac gluten/wheat sensitivity (NCGS)—a currently undefined disorder without validated diagnostic criteria and of unknown prevalence: Position statement of the task force on food allergy of the German Society of Allergology and Clinical Immunology (DGAKI)

**DOI:** 10.1007/s40629-018-0070-2

**Published:** 2018-05-28

**Authors:** Imke Reese, Christiane Schäfer, Jörg Kleine-Tebbe, Birgit Ahrens, Oliver Bachmann, Barbara Ballmer-Weber, Kirsten Beyer, Stephan C. Bischoff, Katharina Blümchen, Sabine Dölle, Paul Enck, Axel Enninger, Isidor Huttegger, Sonja Lämmel, Lars Lange, Ute Lepp, Vera Mahler, Hubert Mönnikes, Johann Ockenga, Barbara Otto, Sabine Schnadt, Zsolt Szepfalusi, Regina Treudler, Anja Wassmann-Otto, Torsten Zuberbier, Thomas Werfel, Margitta Worm

**Affiliations:** 1 Dietary Counseling and Nutrition Therapy with Specialization in Allergology, Ansprenger Str. 19, 80803 Munich, Germany; 2 Outpatient Center of Allergy and Pulmonology (Schwerpunktpraxis Collonaden), Dietary Counseling and Nutrition Therapy, Hamburg, Germany; 3 Outpatient & Clinical Research Center, Allergy and Asthma Center Westend, Berlin, Germany; 4 Division of Allergology, Paul Ehrlich Institute (Federal Institute for Vaccines and Biomedicines), Langen, Germany; 5 Department of Pediatrics, Division of Pulmonology, Immunology and Intensive Care Medicine, Charité University Hospital, Berlin, Germany; 6 Department of Gastroenterology, Hepatology and Endocrinology, Hanover Medical School, Hanover, Germany; 7 Allergy Unit, Department of Dermatology, University Hospital Zurich, Zurich, Switzerland; 8 Institute of Clinical Nutrition, University of Hohenheim, Stuttgart, Germany; 9 Department of Pediatrics, Division of Allergy, Pulmonology and Cystic Fibrosis, University Hospital Frankfurt, Frankfurt am Main, Germany; 10 Department of Dermatology, Venereology and Allergology, Allergy Center Charité (ACC), Charité University Hospital, Berlin, Germany; 11 Department of Internal Medicine VI, Psychosomatic Medicine and Psychotherapy, University Hospital Tübingen, Tübingen, Germany; 12 Derpartment of General and Special Pediatrics, Olga Hospital (Klinikum Stuttgart), Stuttgart, Germany; 13 Pediatric Allergology and Pulmonology, Department of Pediatrics and Adolescent Medicine, Salzburg State Clinics, Paracelsus Medical Private University, Salzburg, Austria; 14 Patient Support Group: Deutscher Allergie- und Asthmabund, DAAB, German Allergy and Asthma Association, Mönchengladbach, Germany; 15 Department of Pediatrics, St. Marien Hospital, Bonn, Germany; 16 Respiratory Medicine and Allergology Outpatient Center Dr. Lepp, Buxtehude, Germany; 17 Department of Dermatology, Faculty of Medicine, Friedrich Alexander University Erlangen-Nürnberg, Erlangen, Germany; 18 Department of Internal Medicine and Institute of Neurogastroenterology, Martin-Luther Hospital, Berlin, Germany; 19 Department of Gastroenterology, Endocrinology and Clinical Nutrition, Klinikum Bremen Mitte, Bremen, Germany; 20 Institute for Medical Education, University Hospital, Ludwig Maximilian University of Munich, Munich, Germany; 21 Pediatric Pulmonology, Allergy and Endocrinology, Department of Pediatrics and Adolescent Medicine, Medical University of Vienna, Vienna, Austria; 22 Department of Dermatology, Venereology and Allergology, Interdisciplinary Center of Allergology, University Medical Center Leipzig, Leipzig, Germany; 23 Dermatology Outpatient Center Hamburg-Alstertal, Hamburg, Germany; 24 Department of Dermatology, Allergology and Venereology, Department of Immunodermatology and Experimental Allergology, Hanover Medical School (MHH), Hanover, Germany

**Keywords:** Gluten-free, Self-diagnosis, Nocebo effects, Placebo effects, Irritable bowel syndrome

## Abstract

Within the last decade, non-celiac gluten/wheat sensitivity (NCGS) has been increasingly discussed not only in the media but also among medical specialties. The existence and the possible triggers of NCGS are controversial. Three international expert meetings which proposed recommendations for NCGS were not independently organized and only partially transparent regarding potential conflicts of interest of the participants. The present position statement reflects the following aspects about NCGS from an allergist’s and nutritionist’s point of view: (A) Validated diagnostic criteria and/or reliable biomarkers are still required. Currently, this condition is frequently self-diagnosed, of unknown prevalence and non-validated etiology. (B) Gluten has not been reliably identified as an elicitor of NCGS because of high nocebo and placebo effects. Double-blind, placebo-controlled provocation tests are of limited value for the diagnosis of NCGS and should be performed in a modified manner (changed relation of placebo and active substance). (C) Several confounders hamper the assessment of subjective symptoms during gluten-reduced or gluten-free diets. Depending on the selection of food items, e.g., an increased vegetable intake with soluble fibers, diets may induce physiological digestive effects and can modify gastrointestinal transit times independent from the avoidance of gluten. (D) A gluten-free diet is mandatory in celiac disease based on scientific evidence. However, a medically unjustified avoidance of gluten may bear potential disadvantages and risks. (E) Due to a lack of diagnostic criteria, a thorough differential diagnostic work-up is recommended when NCGS is suspected. This includes a careful patient history together with a food-intake and symptom diary, if necessary an allergy diagnostic workup and a reliable exclusion of celiac disease. We recommend such a structured procedure since a medically proven diagnosis is required before considering the avoidance of gluten.

## Introduction

Non-celiac gluten sensitivity (NCGS) or non-celiac wheat sensitivity is an increasingly discussed disorder. The mechanism is unknown and reliable biomarkers for diagnosis are lacking. Whether it is a specific disease entity and which wheat component is the responsible trigger is a long-running controversy [1–6]. Reported symptoms may be caused by undiagnosed celiac disease, variants of irritable bowel syndrome (IBS), or other undiagnosed functional disorders of the gut [7, 8]. Thus, individuals reporting gastrointestinal (GI) symptoms after wheat consumption may be wrongly characterized with NCGS. Three international expert meetings concerning NCGS have taken place [9–11]. Those meetings were not organized independent of “interested parties” and findings did not convincingly exclude possible conflict of interest of the participants and/or sponsors. From an allergist’s point of view, the diagnostic algorithm proposed during the third expert meeting is inappropriate for the diagnosis of NCGS [11]. The following issues will be discussed:

1. Absence of validated diagnostic criteria and/or suitable biomarkers, frequent self-diagnosis, undocumented prevalence and unconfirmed etiology of reported symptoms.
2. No reliable identification of gluten as trigger of NCGS during controlled food challenges due to an *a priori* bias of the subject toward experiencing symptoms.
3. Several variables confounding the evaluation of subjective symptoms during gluten-reduced and/or -free diet.
4. Potential disadvantages and risks will prevail in case of medically unjustified gluten avoidance.
5. Proposed diagnostic procedure in suspected NCGS.

### Issue 1

*Absence of validated diagnostic criteria and/or suitable biomarkers, frequent self-diagnosis, undocumented prevalence and unconfirmed etiology of reported symptoms.* As validated diagnostic criteria are lacking to date, the prevalence of NCGS cannot be assessed. Recent survey results are based primarily on self-diagnosis and reveal how many people think that they are affected rather than proving actual prevalence [12–14]. Moreover, the exclusion of other diseases such as IBS, celiac disease or functional GI disorders has not been systematically evaluated in published surveys and studies [4, 7, 8, 15–17]. Without an appropriate differential diagnosis these data should be interpreted with caution [18]. A recent study in individuals reporting wheat sensitivity suggests a compromised intestinal epithelial barrier as cause for a systemic immune activation [19]. The identification of possible triggers was not the aim of the study. The authors consider their findings only as a basis for further research.

### Issue 2

*No reliable identification of gluten as trigger of NCGS during controlled food challenges due and an* a priori *bias of the subject toward experiencing symptoms.* Results from various studies with food challenges of classical double-blind, placebo-controlled (DBPCFC) design reveal that only a minority of individuals suspected of suffering from NCGS are able to correctly identify gluten as trigger [20–23]. It cannot be excluded that positive test results are triggered by the expectation of symptoms rather than gluten as a true elicitor [5, 20–22]. This postulate is supported by the observation that most patients react comparably to actual gluten and placebo [20–22]. In order to mitigate the expectations of a patient, the number of placebo challenges can be increased [24, 25]. Such an approach may identify true gluten responders better than the proposed recommendation to increase the number of gluten challenges [11]. We recommend a ratio of placebo to active of at least 2:1 in controlled challenges. This approach has been successfully utilized in a current study, determining that the majority of patients with suspected NCGS cannot identify gluten as trigger of their symptoms [26].

### Issue 3

*Several variables confounding the evaluation of subjective symptoms during gluten-reduced and/or -free diet.* A gluten-reduced diet can, according to food selection (i. e., if rich in vegetables with soluble fiber), induce physiological digestive effects and alter intestinal transit time independently of gluten content. Therefore, certain food components such as soluble fiber are supposed to elicit a therapeutic effect. Hence, patients may benefit from a gluten-free diet by changing food composition and thereby inducing physiological digestive effects and, thus, altering intestinal transit time physiologically rather than by eliminating gluten [21]. A temporary gluten reduction, but not total gluten avoidance is recommended in the German IBS guideline [27]. As mentioned therein, IBS-afflicted individuals can benefit from a change of fiber quality. Optimal benefit can be achieved if soluble fiber, such as those in psyllium husks and certain vegetables, are increased in parallel to a reduction of cereal fiber [27]. Thus, IBS patients will benefit from their food selection in favor of soluble fiber but not from gluten avoidance. This view is supported by the observation made in several studies that many individuals benefit from a diet free from gluten while only a minority identified gluten in a DBPCFC [6, 20–23, 26]. Apart from gluten, many other potential triggers are discussed, such as fructans, amylase-trypsin inhibitors (ATIs), etc. [3, 4, 28].

### Issue 4

*Potential disadvantages and risks will prevail in case of medically unjustified gluten avoidance.* A strict gluten-free diet is mandatory in confirmed celiac disease. In contrast, potential disadvantages and risks exist in the case of self-diagnosis without professional dietetic support [27, 29, 30]. Risks of gluten-free diet without a medically proven indication are as follows:

- masking of undiagnosed celiac disease [17, 18],
- triggering of an eating disorder, such as Orthorexia nervosa [15],
- eliciting or worsening of constipation, potentially causing rectal diseases [31, 32], and
- increased risk of dyslipidemia [33].

Furthermore, there are known disadvantages of a gluten-free diet regarding

- inadequate nutrition [29, 30],
- impaired quality of life [34],
- higher food costs [35], and
- potential heavy metal contamination [36, 37].

Therefore, a recommendation for a temporarily limited gluten reduction (as mentioned in the German IBS guideline) is reasonable. In contrast, a recommendation for a gluten-free diet without medically proven diagnosis (celiac disease) is not currently warranted.

### Issue 5

**Fig. 1:**
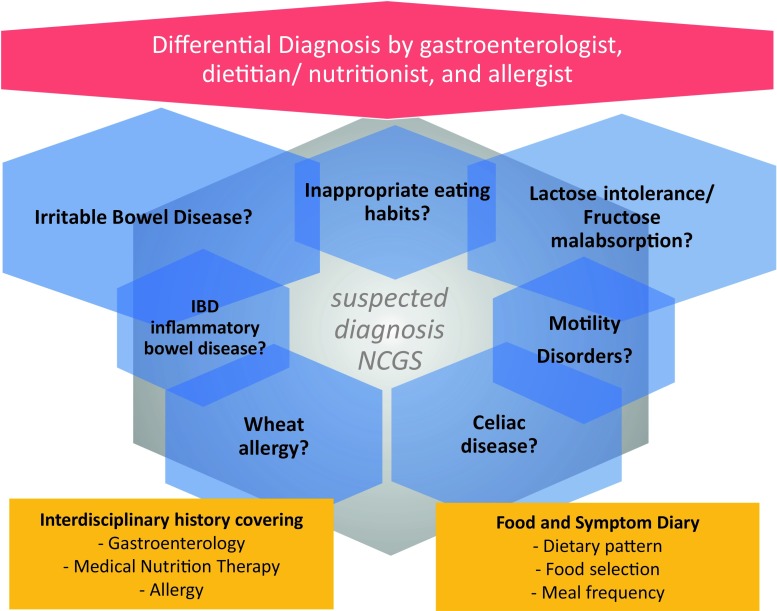
Important differential diagnoses in case of suspected non-celiac gluten/wheat sensitivity (NCGS) cover various disorders, including functional or inflammatory bowel diseases, allergies, enzyme deficiencies/malabsorption and autoimmune diseases

*Proposed diagnostic procedure in suspected NCGS.* Lacking viable criteria, a proven diagnosis of NCGS is not possible. A thorough differential diagnostic work-up is mandatory (Fig. 1), which includes the following: a comprehensive and interdisciplinary patient history in combination with the evaluation of a food intake/symptom diary; if justified an allergy work-up; and a definitive exclusion of celiac disease—to be meaningful, gluten must be part of the diet for at least three months in sufficiently high amounts (15–20 g gluten per day, equaling 4–5 slices of bread).

## Conclusion

Without a confirmed diagnosis, a gluten-free diet is unjustified and not recommended. Patients who intend to continue to restrict their diet despite the recommendation should be encouraged to seek professional nutritional counselling.

## Abbreviations

ATI: Amylase-trypsin inhibitors
DBPCFC: Double-blind, placebo-controlled food challenge
DGAKI: German Society of Allergology and Clinical Immunology
IBS: Irritable bowel syndrome
NCGS: Non-celiac gluten/wheat sensitivity
